# 造血干细胞移植后肝窦隙阻塞综合征诊断与治疗中国专家共识（2022年版）

**DOI:** 10.3760/cma.j.issn.0253-2727.2022.03.001

**Published:** 2022-03

**Authors:** 中华医学会血液学分会

肝窦隙阻塞综合征（sinusoidal obstruction syndrome, SOS）也称肝静脉闭塞症（veno-occlusive disease, VOD），多发生于摄入某些毒性生物碱、高剂量放/化疗和器官移植的患者，是一种可危及生命的严重疾病。造血干细胞移植（HSCT）患者是SOS最主要的发病人群，SOS也是移植早期的重要合并症和死亡原因之一。本共识在参考该领域国外指南/共识的基础上，纳入国内的主要研究成果和临床经验，结合现时国情，为各移植单位SOS规范化诊疗提供指导性意见。

一、定义和流行病学

HSCT后SOS是指HSCT后早期发生的、预处理相关肝毒性导致的一类主要表现为黄疸、液体潴留、肝肿大等特征的临床综合征，重症患者病死率可高达80％[1]–[3]。

因患者特征、预处理方案、移植中心经验、诊断标准等差异，SOS发生率在不同研究中的差异较大。一项荟萃分析显示中位发生率为13.7％（0～62.3％）[4]。综合来看，自体HSCT（auto-HSCT）后发生率为3.1％～8.7％，异基因HSCT（allo-HSCT）为8.9％～14.0％[4]–[8]。儿童HSCT患者发生率略高于成人[9]–[12]。国内广西医科大学附属第一院报道allo-HSCT后SOS发生率为7.4％[13]。近年来发生率及严重程度总体有所下降，但某些药物（如CD33、CD22等单抗）的应用、增加强度预处理、二次移植等在一定程度上会增加患者发病风险。

二、发病机制

SOS的发病机制尚未明确，目前认为预处理方案相关肝毒性为首要病因。白消安（BU）、环磷酰胺（CTX）、全身放疗（TBI）等对窦隙内皮细胞（SEC）和肝细胞的毒性损伤是SOS发生的直接原因，肝小叶中心区域（肝腺泡3区）因缺乏谷胱甘肽（GSH）而更易发生损伤[14]–[16]。除预处理外，组织损伤产生的细胞因子、药物［钙调神经磷酸酶抑制剂（CNI）、造血生长因子、抗体药物等］、异源反应性T细胞、某些GSHS-转移酶相关基因突变、内源微生物代谢物的迁移及造血植入等也可导致或加重SEC损伤[17]–[24]。SEC损伤导致内皮细胞屏障破坏，窦壁完整性丧失，红细胞渗至狄氏（Disse）腔，引起内皮细胞分离，造成肝窦隙阻塞；此外，凝血-纤溶系统失衡可致微血栓形成，加重小叶中心静脉阻塞，最终形成窦后性门静脉高压。发展至重症者小叶中心坏死、纤维化，肝功能衰竭。鉴于内皮损伤在发病机制中的中心地位，也有学者将SOS纳入HSCT相关内皮损伤综合征范畴[25]–[26]。

三、危险因素

SOS发病危险因素一般分为患者相关和移植相关两类。前者主要包括：年龄、体能状况、移植前肝病史/肝功能异常、疾病进展状态、地中海贫血、铁过载、腹部放疗史、应用吉妥珠单抗（Gemtuzumabozogamicin）或奥加伊妥珠单抗（Inotuzumabozogamicin）[27]–[29]。后者主要包括：allo-HSCT（相比auto-HSCT）、HLA不合/单倍型移植、二次移植、移植物非去T细胞、含BU或TBI预处理、氟达拉滨、CNI联合西罗莫司预防GVHD等。

识别危险因素或构建前瞻性风险评估模型有助于SOS的早期预测和预防[30]。

四、诊断、分级及鉴别诊断

典型SOS多发生于HSCT后21 d内，迟发型可发生于21 d后。可隐匿发病，也可急骤进展。主要临床表现包括右上腹压痛、黄疸、痛性肝肿大、腹水、体重增加、水肿等。实验室检查可见高胆红素血症、转氨酶升高、难以解释的血小板减少等。影像学（推荐多普勒超声）检查可发现肝肿大、腹水、胆囊壁水肿、肝/门静脉血流减慢或反向血流、门静脉增宽等[31]–[32]。轻症患者呈自限性，重症者可出现肾、肺、心脏等多器官功能衰竭（MOF），预后凶险。

肝组织活检病理是诊断金标准，但在移植早期实施出血风险大，不常规推荐。有经验的单位可选择经颈静脉肝活检或测量肝静脉压力梯度（HVPG）辅助诊断。近年以瞬时弹性成像技术（TE）进行肝硬度检测（LSM），预测和诊断的敏感性及特异性较高[33]–[35]。

目前尚无具有预测或诊断意义的生物学标志物。纤溶酶原激活物抑制物-1（PAI-1）等血凝标志物有一定的预测价值，内皮细胞损伤及炎症标志物尚在探索中[36]–[40]，不推荐常规检测。

SOS临床诊断多依据修订的西雅图（Seattle）标准[41]或巴尔的摩（Baltimore）标准[42]。2016年欧洲骨髓移植学会（EBMT）提出的SOS标准[3]具有较好的实用性，本共识推荐使用该标准，也可与前述2个标准并行使用。各诊断标准见表1。

**表1:** 修订的西雅图、巴尔的摩和欧洲骨髓移植学会（EBMT）肝窦隙阻塞综合征（SOS）诊断标准

标准	描述
修订的西雅图标准	HSCT后20 d内出现至少2条下述表现：胆红素>2 mg/dl，肝肿大伴右上腹痛，液体潴留致体重增加≥2％基线体重
巴尔的摩标准	HSCT后21 d内胆红素>2 mg/dl，同时至少符合2条下述表现：痛性肝肿大，体重增加≥5％，腹水
EBMT标准	经典型SOS：HSCT后21 d内胆红素>2 mg/dl，同时至少符合2条下述表现：痛性肝肿大，体重增加≥5％，腹水迟发型SOS：HSCT后21 d后出现经典型SOS或病理学证实的SOS，或≥2条经典型标准且同时具备超声或血液动力学证据

注：HSCT：造血干细胞移植

儿童SOS的EBMT诊断标准[10]：无发生时间限定，至少满足2条下述表现：①难以解释的消耗性血小板减少和无效输注；②即使应用利尿剂，仍有难以解释的连续3 d体重增加或体重增加>5％基线值；③高于基线值的肝脏肿大（建议影像学确认）；④高于基线值的腹水（建议影像学确认）；⑤连续3 d胆红素高于基线值，或72 h内胆红素≥2 mg/dl。值得注意的是，16％～20％的儿童SOS为迟发型，近30％无黄疸表现[43]–[44]。

本共识推荐采用美国血液学会SOS分级标准[45]（表2）及EBMT分级标准[3]（表3）进行严重程度分级。

**表2:** 美国血液学会肝窦隙阻塞综合征（SOS）分级标准

分级	病情进展	胆红素（mg/dl）	转氨酶	体重增幅	血肌酐
轻度	慢，（>6～7 d）	2～3	<3倍正常值上限	2.0％	正常
中度	快，（4～5 d）	>3～5	3～5倍正常值上限	2.1％～5.0％	<2倍基线值
重度	迅速，（2～3 d）	>5	>5倍正常值上限	>5.0％	≥2倍基线值

注：上述标准符合≥2条即可确认相应严重程度

**表3:** 欧洲骨髓移植学会肝窦隙阻塞综合征分级标准

分级	首次症状出现时间（d）	胆红素（mg/dl）	胆红素变化	转氨酶	体重增加	血肌酐
轻度	>7	2～<3		≤2倍正常值上限	<5％	<1.2倍移植前基线值
中度	5～7	3～<5		>2～5倍正常值上限	5％～<10％	1.2～<1.5倍移植前基线值
重度	≤4	5～<8	48 h内倍增	>5～8倍正常值上限	5％～<10％	1.5～<2倍移植前基线值
极重度	任何时间	≥8		>8倍正常值上限	≥10％	≥2倍移植前基线值或出现其他多器官功能衰竭表现

注：当符合不同分类下的2条标准时，应归类于比较严重的分类中

SOS需要与以下疾病鉴别：肝脏急性GVHD、病毒性肝炎、药物性肝损伤、毛细血管渗漏综合征（CLS）、移植相关血栓性微血管病（TA-TMA）等，鉴别要点见表4。

**表4:** 肝窦隙阻塞综合征（SOS）的鉴别诊断

疾病	临床特征	辅助检查
肝脏急性GVHD	淤胆性肝损伤为主，伴或不伴肝酶增高，多伴有皮肤和（或）肠道急性GVHD，孤立的肝脏急性GVHD较少见。少见痛性肝肿大及钠水潴留	无SOS超声特征
病毒性肝炎	HBV再激活：HBV感染史，急性肝炎表现伴病毒载量显著增高，排除其他病因。其他病毒感染：HCV、HEV、HSV、CMV、EBV、ADV、HHV-6等。少见痛性肝肿大、钠水潴留	同上
药物性肝损伤	评估可疑药物与肝损伤的相关程度，药物应用与肝损伤的时间关系，停药后反应等。重点关注CNI、抗真菌药物和部分抗生素。无痛性肝肿大、钠水潴留	同上
毛细血管渗漏综合征	可为植入综合征表现，也可发生于感染及CNI、细胞因子应用后。浮肿、非心源性肺水肿、低蛋白血症、对利尿剂反应差。少见黄疸、肝肿大	可有腹水征
移植相关血栓性微血管病	难以解释的肾功能和（或）中枢神经系统异常，微血管病性溶血，破碎红细胞，高血压，血浆sC5b-9增高等。少见肝受累	部分患者有腹水征

注：GVHD：移植物抗宿主病；HBV：乙型肝炎病毒；HCV：丙型肝炎病毒；HEV：戊型肝炎病毒；HSV：单纯疱疹病毒；CMV：巨细胞病毒；EBV：EB病毒；ADV：腺病毒；HHV-6：人类疱疹病毒6型；CNI：钙调神经磷酸酶抑制剂；sC5b-9：可溶性补体膜攻击复合物

五、预防

1. 一般原则：避免SOS危险因素，包括祛铁治疗、避免肝炎活动期进行HSCT、预处理方案调整（减低强度、药代动力学指导BU用药、分次TBI等）、避免合用肝毒性药物、警惕某些药物应用（CD33/CD22单抗等）增加SOS风险；液体平衡管理（避免超负荷，同时维持有效血容、避免肾灌注不足）；HSCT后早期应监测体重、腹围等变化。

2. 预防药物：①熊去氧胆酸（UDCA）：随机对照临床试验（RCT）及荟萃分析显示UDCA可降低HSCT后SOS发生率[46]–[49]。部分研究未能观察到上述结果，但发现Ⅲ/Ⅳ度肝脏急性GVHD发生率明显下降，1年总生存（OS）率更优[50]。目前，UDCA在国内外已得到普遍应用[25],[51]–[52]。推荐用法：UDCA 12～15 mg·kg^−1^·d^−1^，移植前开始服用，移植后100 d停药。②普通肝素或低分子量肝素：临床应用和试验研究较多，RCT及荟萃分析（包括儿童及成人）结论不一[7],[53]–[57]，国内应用较多。前列腺素E1（PGE1）：相关RCT研究缺少一致性结论，国内应用较多。③中成药：复方丹参、复方川穹嗪等，国内部分移植中心有应用经验。④去纤苷（DF）：提取自猪肠黏膜的一种单链多聚脱氧核苷酸复合物，机制尚未完全阐明。初步发现具有保护内皮、恢复血栓-纤溶平衡、抗凝及调节血小板活性等作用，不显著增加出血风险。DF是目前国外唯一获批的SOS治疗药物，尚未批准用于预防，但多个预防的RCT研究结果令人鼓舞[9],[58]–[59]。荟萃分析显示，DF预防组SOS发生率显著低于对照组（4.7％对13.7％）[60]。除降低SOS发生率外，DF还可降低SOS相关死亡率及急性GVHD发生率[9],[61]–[62]。推荐用法：DF 6.25 mg/kg，每6 h 1次，每次维持2 h静脉给药，自预处理开始用药，移植后30 d停药。

本共识建议，SOS高危患者，如有条件可选用DF预防，常规预防可选用UDCA、普通或低分子肝素、前列腺素E1及中成药等，也可联合用药，建议各中心根据各自经验选用[63]。鼓励积极开展相关的临床试验研究。

六、治疗

进行严重程度分级有利于分层治疗。约70％的轻症患者经暂停CNI等可疑药物并给予利尿、液体平衡管理等支持治疗即可恢复。暂停CNI时应审慎评估GVHD风险，必要时予糖皮质激素、霉酚酸酯、CD25单抗等药物替代。重度及极重度患者应立即启动特异性治疗。轻、中度患者接受支持治疗，严密观察并根据病情变化及时调整治疗方案，以防病情恶化。

1. 支持治疗：每日监测患者体重、腹围、尿量、出入量等，评估病情及治疗反应。去除可疑诱因，严格管理水钠摄入，利尿，输注白蛋白、血浆或成分血，维持循环血量和肾灌注。胸/腹腔大量积液时，可适度抽液以减轻压迫。低氧状态时给予氧疗或机械通气。必要时镇痛治疗，合并肾功能衰竭时进行血液透析或滤过治疗。重症患者建议转重症监护病房（ICU）或进行多学科会诊（MDT）。

2. 特异性治疗：常用药物包括DF、重组人组织型纤溶酶原激活物（rh-tPA）、糖皮质激素等。

（1）DF：DF是欧美国家唯一批准的重度SOS治疗药物，疗效和安全性已被多个较高质量的临床研究证实。完全缓解（CR）率为25.5％～55.0％，100 d生存率为38.2％～58.9％（不伴MOF者达71.0％），儿童疗效优于成人，主要不良事件为出血（肺、消化道）[64]–[67]。一项纳入140例SOS患者的上市后Ⅳ期研究结果显示，DF治疗后100 d生存率为58％，其中重度病例生存率为79％，极重度病例为34％[68]。推荐用法：6.25 mg·kg^−1^·h^−1^（2 h静脉滴注），依据治疗反应用药2～3周。有出血风险患者，可根据经验酌情减量。获得CR或发生严重出血时，可停药观察。

（2）rh-tPA：属丝氨酸蛋白酶，与纤维蛋白结合后，诱导纤溶酶原转化为纤溶酶，降解纤维蛋白，发挥溶栓活性。较早期的国外指南将其列为不能获得DF时的可选择药物之一，后基于较高的严重出血风险（近30％）而不再推荐[25]。近年国内陈峰等以低剂量rh-tPA（10 mg/d）为主方案治疗16例HSCT后重度/极重度SOS，CR率及100 d生存率均达到75％，无严重出血相关死亡[69]–[70]。

（3）糖皮质激素：早期应用有一定疗效。甲泼尼龙（MP）0.5 mg/kg，每日2次，反应率为63％，100 d生存率为58％[71]。Myers等[72]应用MP治疗儿童SOS（500 mg/m^2^，每日2次），反应率为66.7％。应用时应警惕增加感染风险。

（4）其他：对治疗无反应、进展的SOS患者，如有条件，可尝试经颈静脉肝内门体静脉分流术（TIPS）、肝移植等挽救治疗。

共识建议采取分层治疗策略，需特异性治疗的患者在支持治疗基础上可加用DF。目前DF尚未在国内上市，各中心可根据各自经验选择低剂量rh-tPA、糖皮质激素等治疗，鼓励开展相关的临床试验研究。

## References

[1] Tewari P, Wallis W, Kebriaei P (2017). Manifestations and management of veno-occlusive disease/sinusoidal obstruction syndrome in the era of contemporary therapies[J]. Clin Adv Hematol Oncol.

[2] Mohty M, Malard F, Abecassis M (2015). Sinusoidal obstruction syndrome/veno-occlusive disease: current situation and perspectives—a position statement from the European Society for Blood and Marrow Transplantation (EBMT)[J]. Bone Marrow Transplant.

[3] Mohty M, Malard F, Abecassis M (2016). Revised diagnosis and severity criteria for sinusoidal obstruction syndrome/veno-occlusive disease in adult patients: a new classification from the European Society for Blood and Marrow Transplantation[J]. Bone Marrow Transplant.

[4] Coppell JA, Richardson PG, Soiffer R (2010). Hepatic veno-occlusive disease following stem cell transplantation: incidence, clinical course, and outcome[J]. Biol Blood Marrow Transplant.

[5] Tsirigotis PD, Resnick IB, Avni B (2014). Incidence and risk factors for moderate-to-severe veno-occlusive disease of the liver after allogeneic stem cell transplantation using a reduced intensity conditioning regimen[J]. Bone Marrow Transplant.

[6] Carreras E, Díaz-Beyá M, Rosiñol L (2011). The incidence of veno-occlusive disease following allogeneic hematopoietic stem cell transplantation has diminished and the outcome improved over the last decade[J]. Biol Blood Marrow Transplant.

[7] Carreras E, Bertz H, Arcese W (1998). Incidence and outcome of hepatic veno-occlusive disease after blood or marrow transplantation: a prospective cohort study of the European Group for Blood and Marrow Transplantation[J]. Blood.

[8] Coppell JA, Richardson PG, Soiffer R (2010). Hepatic veno-occlusive disease following stem cell transplantation: incidence, clinical course, and outcome[J]. Biol Blood Marrow Transplant.

[9] Corbacioglu S, Cesaro S, Faraci M (2012). Defibrotide for prophylaxis of hepatic veno-occlusivedisease in paediatric haemopoietic stem-cell transplantation: an openlabel, phase 3, randomised controlled trial[J]. Lancet.

[10] Corbacioglu S, Carreras E, Ansari M (2018). Diagnosis and severity criteria for sinusoidal obstruction syndrome/veno-occlusive disease in pediatric patients: a new classification from the European society for blood and marrow transplantation[J]. Bone Marrow Transplant.

[11] Barker CC, Butzner JD, Anderson RA (2003). Incidence, survivaland risk factors for the development of veno-occlusive disease in pediatrichematopoietic stem cell transplant recipients[J]. Bone Marrow Transplant.

[12] Cesaro S, Pillon M, Talenti E (2005). A prospective survey on incidence, risk factors and therapy of hepatic venoocclusive disease in children after hematopoietic stem cell transplantation[J]. Haematologica.

[13] 覃 春捷, 刘 练金, 章 忠明 (2018). 造血干细胞移植后肝静脉闭塞病的临床分析[J]. 中华内科杂志.

[14] Hassan M, Ljungman P, Ringdén O (2000). The effect of busulphan on the pharmacokinetics of cyclophosphamide andits 4-hydroxy metabolite: time interval influence on therapeutic efficacyand therapy-related toxicity[J]. Bone Marrow Transplant.

[15] Zeng L, Jia L, Xu S (2010). Vascular endotheliumchanges after conditioning in hematopoietic stem cell transplantation:role of cyclophosphamide and busulfan[J]. Transplant Proc.

[16] Carreras E, Diaz-Ricart M (2011). The role of the endothelium in the shortterm complications of hematopoietic SCT[J]. Bone Marrow Transplant.

[17] Palomo M, Diaz-Ricart M, Carbo C (2009). The release of soluble factors contributing to endothelialactivation and damage after hematopoietic stem cell transplantation isnot limited to the allogeneic setting and involves several pathogenicmechanisms[J]. Biol Blood Marrow Transplant.

[18] Eissner G, Multhoff G, Holler E (1998). Influence of bacterial endotoxin on theallogenicity of human endothelial cells[J]. Bone Marrow Transplant.

[19] Fuste B, Escolar G, Marin P (2004). G-CSF increases the expression of VCAM-1 on stromal cells promoting theadhesion of CD34^+^ hematopoietic cells: studies under flow conditions[J]. Exp Hematol.

[20] Mercanoglu F, Turkmen A, Kocaman O (2004). Endothelial dysfunction in renal transplant patientsis closely related to serum cyclosporine levels[J]. Transplant Proc.

[21] Zoja C, Furci L, Ghilardi F (1986). Cyclosporininduced endothelial cell injury[J]. Lab Invest.

[22] Biedermann BC, Sahner S, Gregor M (2002). Endothelial injury mediated by cytotoxic T lymphocytes and loss ofmicrovessels in chronic graft versus host disease[J]. Lancet.

[23] Srivastava A, Poonkuzhali B, Shaji RV (2004). Glutathione S-transferase M1 polymorphism: a risk factor forhepatic venoocclusive disease in bone marrow transplantation[J]. Blood.

[24] Bonifazi F, Barbato F, Ravaioli F (2020). Diagnosis and treatment of VOD/SOS after allogeneichematopoietic stem celltransplantation[J]. Front Immunol.

[25] Carreras E, Dufour C, Mohty M (2019). The EBMT Handbook[M].

[26] Carreras E, Diaz-Ricart M (2011). The role of the endothelium in the shortterm complications of hematopoietic SCT[J]. Bone Marrow Transplant.

[27] Kantarjian HM, DeAngelo DJ, Advani AS (2017). Hepatic adverse event profile of inotuzumab ozogamicin in adult patients with relapsed or refractory acute lymphoblastic leukaemia: results from the open-label, randomised, phase 3 INO-VATE study[J]. Lancet Haematol.

[28] Kantarjian HM, Vandendries E, Advani AS (2016). Inotuzuma bozogamicinfor acute lymphoblastic leukemia[J]. N Engl J Med.

[29] Kebriaei P, Cutler C, de Lima M (2018). Management of important adverse events associated with inotuzuma bozogamicin: expert panel review[J]. Bone Marrow Transplant.

[30] Strouse C, Zhang Y, Zhang MJ (2018). Risk score for the development of veno-occlusive disease afterallogeneic hematopoietic cell transplant[J]. Biol Blood Marrow Transplant.

[31] Chan SS, Colecchia A, Duarte RF (2020). Imaging in hepatic veno-occlusive disease/sinusoidal obstruction syndrome[J]. Biol Blood Marrow Transplant.

[32] Nishida M, Kahata K, Hayase E (2018). Novel ultrasonographic scoring system of sinusoidal obstruction syndrome after hematopoietic stem cell transplantation[J]. Biol Blood Marrow Transplant.

[33] Colecchia A, Marasco G, Ravaioli F (2017). Usefulness of liver stiffness measurement in predicting hepatic veno-occlusive disease development in patients who undergo HSCT[J]. Bone Marrow Transplant.

[34] Colecchia A, Ravaioli F, Sessa M (2019). Liver stiffness measurement allows early diagnosis of veno-occlusive disease/sinusoidal obstruction syndrome in adult patients who undergo hematopoietic stem cell transplantation: results from a monocentric prospective study[J]. Biol Blood Marrow Transplant.

[35] Reddivalla N, Robinson AL, Reid KJ (2020). Using liver elastography to diagnose sinusoidal obstruction syndrome in pediatric patients undergoing hematopoetic stem cell transplant[J]. Bone Marrow Transplant.

[36] Corbacioglu S, Jabbour EJ, Mohty M (2019). Risk factors for development of and progression of hepatic veno-occlusive disease/sinusoidal obstruction syndrome[J]. Biol Blood Marrow Transplant.

[37] Je-Hwan Lee, Kyoo-Hyung Lee, Jung-Hee Lee (2002). Plasminogen activator inhibitor-1 is an independent diagnostic marker aswell as severity predictor of hepatic veno-occlusive disease afterallogeneic bone marrow transplantation in adults conditioned withbusulphan and cyclophosphamide[J]. Br J Haematol.

[38] Pihusch M, Wegner H, Goehring P (2005). Diagnosis of hepatic veno-occlusive disease by plasminogen activator inhibitor-1 plasma antigen levels: a prospective analysis in 350 allogeneic hematopoietic stem cell recipients[J]. Transplantation.

[39] Sartori MT, Cesaro S, Peruzzo M (2012). Contribution of fibrinolytic tests to the differential diagnosisof veno-occlusive disease complicating pediatric hematopoieticstem cell transplantation[J]. Pediatr Blood Cancer.

[40] Mega A, Gastl G, Cesaro S (2017). New insights into sinusoidal obstruction syndrome[J]. Intern Med J.

[41] McDonald GB, Hinds MS, Fisher LD (1993). Veno-occlusive disease of the liver and multiorgan failure after bone marrow transplantation: a cohort study of 355 patients[J]. Ann Intern Med.

[42] Jones RJ, Lee KS, Beschorner WE (1987). Venoocclusive disease of the liver following bone marrow transplantation[J]. Transplantation.

[43] Kernan NA, Grupp S, Smith AR (2018). Final results from a defibrotide treatment-IND study for patients with hepatic veno-occlusive disease/sinusoidal obstruction syndrome[J]. Br J Haematol.

[44] Myers KC, Dandoy C, EI-Bietar J (2015). Veno-occlusive disease of the liver in the absence of elevation in bilirubin in pediatric patients after hematopoietic stem cell transplantation[J]. Biol Blood Marrow Transplant.

[45] Chao N (2014). How I treat sinusoidal obstruction syndrome[J]. Blood.

[46] Dalle JH, Giralt SA (2016). Hepatic veno-occlusive disease after hematopoietic stem cell transplantation: risk factors and stratification, prophylaxis, and treatment[J]. Biol Blood Marrow Transplant.

[47] Essell JH, Schroeder MT, Harman GS (1998). Ursodiol prophylaxis against hepatic complications of allogeneic bone marrow transplantation. A randomized, double-blind, placebo-controlled trial[J]. Ann Intern Med.

[48] Ohashi K, Tanabe J, Watanabe R (2000). The Japanese multicenter open randomized trial of ursodeoxycholic acid prophylaxis for hepatic veno-occlusive disease after stem cell transplantation[J]. Am J Hematol.

[49] Ruutu T, Eriksson B, Remes K (2002). Ursodeoxycholic acid for the prevention of hepatic complications in allogeneic stem cell transplantation[J]. Blood.

[50] Tay J, Tinmouth A, Fergusson D (2007). Systematic review of controlled clinical trials on the use of ursodeoxycholicacidfor the prevention of hepatic veno-occlusive disease in hematopoietic stem cell transplantation[J]. Biol Blood Marrow Transplant.

[51] Dignan FL, Wynn RF, Hadzic N (2013). BCSH/BSBMT guideline: diagnosis and management of veno-occlusive disease (sinusoidal obstruction syndrome) following haematopoietic stem cell transplantation[J]. Br J Haematol.

[52] Bonifazi F, Sica S, Angeletti A (2021). Veno-occlusive diseasein HSCT patients: consensus-based recommendations for risk assessment, diagnosis and management by the GIMTO group[J]. Transplantation.

[53] Marsa-Vila L, Gorin NC, Laporte JP (1991). Prophylactic heparin does not prevent liver veno-occlusive disease following autologous bone marrow transplantation[J]. Eur J Haematol.

[54] Bearman SI, Hinds MS, Wolford JL (1990). A pilot study of continuous infusion heparin for the prevention of hepatic veno-occlusive disease after bone marrow transplantation[J]. Bone Marrow Transplant.

[55] Imran H, Tleyjeh IM, Zirakzadeh A (2006). Use ofprophylactic anticoagulation and the risk of hepatic veno-occlusive diseasein patients undergoing hematopoietic stem cell transplantation: a systematicreview and meta-analysis[J]. Bone Marrow Transplant.

[56] Simon M, Hahn T, Ford LA (2001). Retrospective multivariate analysis of hepatic veno-occlusive disease after blood or marrow transplantation: possible beneficial use of low molecular weight heparin[J]. Bone Marrow Transplant.

[57] Forrest DL, Thompson K, Dorcas VG (2003). Low molecular weight heparin for the prevention of hepatic veno-occlusivedisease (VOD/SOS) after hematopoietic stem cell transplantation: aprospective phase II study[J]. Bone Marrow Transplant.

[58] Corbacioglu S, Hönig M, Lahr G (2006). Stem cell transplantation in children with infantile osteopetrosis is associated witha high incidence of VOD/SOS, which could be prevented with defibrotide[J]. Bone Marrow Transplant.

[59] Qureshi A, Marshall L, Lancaster D (2008). Defibrotide in the prevention and treatment of veno-occlusive disease in autologous and allogeneic stem cell transplantation in children[J]. Pediatr Blood Cancer.

[60] Chalandon Y, Roosnek E, Mermillod B (2004). Prevention of veno-occlusive disease with defibrotide after allogeneic stem cell transplantation[J]. Biol Blood Marrow Transplant.

[61] Zhang L, Wang Y, Huang H (2012). Defibrotide for the prevention of hepatic veno-occlusive disease after hematopoietic stem cell transplantation: a systematic review[J]. Clin Transplant.

[62] Soyer N, Gunduz M, Tekgunduz E (2020). Incidence and risk factors for hepatic sinusoidal obstruction syndrome after allogeneic hematopoietic stem cell transplantation: A retrospective multicenter study of Turkish hematology research and education group (ThREG)[J]. Transfus Apher Sci.

[63] 陈 洁, 朱 康尔, 张 涛 (2007). 小剂量肝素预防异基因造血干细胞移植后肝静脉阻塞症[J]. 中华内科杂志.

[64] Richardson PG, Riches ML, Kernan NA (2016). Phase 3 trial of defibrotide for the treatment of severe veno-occlusive disease and multi-organ failure[J]. Blood.

[65] Richardson PG, Murakami C, Jin Z (2002). Muliti-institutional use of defibrotide in 88 patients after stem cell transplantation with severe veno-occlusive disease and multisystem organ failure: response without significant toxicity in a high-risk population and factors predictive of outcome[J]. Blood.

[66] Richardson PG, Soiffer RJ, Antin JH (2010). Defibrotide for the treatment of severe veno-occlusive disease and multiorgan failure after stem cell transplantation: a multicenter,randomized, dose-finding trial[J]. Biol Blood Marrow Transplant.

[67] Corbacioglu S, Carreras E, Mohty M (2016). Defibrotide for the treatment of hepatic veno-occlusive disease: finalresults from the international compassionate-use program[J]. Biol BloodMarrow Transplant.

[68] Mohty M, Labopin M, Lebon D (2019). Efficacy and safety of defibrotide in the treatment of hepatic veno-occlusivedisease/sinusoidal obstruction syndrome following hematopoietic stem celltransplantation: interim results from the defifrance study[J]. Bone MarrowTransplant.

[69] 吴 德沛 (2016). 我如何治疗造血干细胞移植后重度肝窦隙阻塞综合征[J]. 中华血液学杂志.

[70] Chen Feng, Zhang Yanmin, Wu Depei (2017). “Defibrotide-Free” regimen as an alternative management of late-onset severe sinusoidal obstruction syndrome after allogeneic hematopoietic stem cell transplantation shows impressive responses[J]. Blood.

[71] AI Beihany A, AI Omar H, Sahovic E (2008). Successful treatment of hepatic veno-occlusive disease after myeloablative allogeneic hematopoietic stem cell transplantation by early administration of a short course of methylprednisolone[J]. Bone Marrow Transplant.

[72] Myers KC, Lawrence J, Marsh RA (2013). High-dose methylprednisolone for veno-occlusive disease of liver in pediatric hematopoietic stem cell transplantation recipients[J]. Biol Blood Marrow Transplant.

